# Challenges in the clinical advancement of cell therapies for Parkinson’s disease

**DOI:** 10.1038/s41551-022-00987-y

**Published:** 2023-01-12

**Authors:** Sophie Skidmore, Roger A. Baker

**Affiliations:** 1 WT-MRC Cambridge Stem cell institute, Jeffrey Cheah Biomedical Centre Cambridge Biomedical Campus, Puddicombe Way, Cambridge CB2 0AW; 2 John van Geest Centre for Brain Repair, E.D. Adrian building, Forvie site, Robinson way, Cambridge, CB2 0PY

## Abstract

Cell therapies as potential treatments for Parkinson’s disease first gained traction in the 1980s, owing to the clinical success of trials that used transplants of foetal midbrain dopaminergic tissue. However, the poor standardization of the tissue for grafting, and constraints on its availability and ethical use, have hindered this treatment strategy. Recent advances in stem cell technologies and in the understanding of the development of dopaminergic neurons have enabled preclinical advancements of promising stem cell therapies. However, to advance to the clinic, challenges in the appropriate levels of safety screening and optimization needed for the cell products as well as in the scalability of their manufacturing will need to be overcome. In this Review Article, we discuss how current challenges, pertaining to cell source, functional and safety testing, manufacturing and storage, and clinical-trial design, are being addressed to advance the translational and clinical development of cell therapies for Parkinson’s disease.

For a subset of neurological diseases that can be characterized by the localized dysfunction of a specific cell type and neurotransmitter, cell-based therapies offer a reparative alternative to the conventional treatment of symptoms. This is the case of Parkinson’s disease (PD), which is characterized by the selective degeneration of nigrostriatal dopaminergic neurons, and is therefore an obvious candidate for dopamine cell-based therapies. Such therapies have been tested for many years with different cell sources, but the only one that has shown consistent promise to date uses tissue from the developing human foetal ventral mesencephalon. However, the use of foetal tissue is hindered by ethical considerations and by major logistical constraints. Also, this treatment strategy does not allow for the standardization of the cell product, as every patient would have a graft derived from their own unique collection of foetal material. Hence, a more robust cell source that could be used to make large numbers of relevant cells for reproducible and reliable grafting is needed. These cells would need to be of the type lost in PD — namely, A9 dopaminergic midbrain progenitors and neurons — and should display characteristic fibre outgrowth, innervation, the release of dopamine, and functional benefits. Moreover, for such a therapy to be considered feasible, economic and reproducible large-scale cell manufacturing is necessary.

In this Review, we discuss the rationale for dopamine cell-based therapies for PD, and the translational and clinical barriers that have been identified. Where appropriate, we briefly refer to other relevant cell therapies and diseases.

## Dopamine cell-based therapies

For 50 years, Levodopa — the precursor to dopamine — has remained the first-line pharmaceutical for the management of PD. Despite its efficacy, chronic Levodopa treatment is associated with long-term side effects, in particular the onset of drug-induced dyskinesia^1^. And its efficacy becomes less reliable with disease progression^2^. In such instances, dopamine receptor agonists and monoamine oxidase inhibitors can be used (separately or together). Alternatively, dopamine receptor agonists and catechol-O-methyltransferase inhibitors can also be used (separately or co-prescribed). And amantadine can be prescribed with any of these combinations to improve the control of the dopaminergic features (motor symptoms) of PD while reducing any Levodopa-induced dyskinesia.

All patients with PD have an array of non-motor comorbidities associated with their condition, which often have a non-dopaminergic basis. The amount of drug necessary to treat these clinical features means that some PD patients can find themselves subject to ever increasingly complex drug regimens, with concomitant side effects. Indeed, about 50% of patients are on more than 12 drugs^3^. These complex dosing regimens in PD patients also bring with them issues of non-adherence^4^ and high costs (estimations of treatment costs have been in the range of £560,000-£1.6 million per 100,000 people per year in the United Kingdom^5^).

To address this, a diverse range of therapeutic options —in addition to cell therapies, gene therapies^6–8^, immunotherapies^9–13^ and pharmacotherapies^14^ — are under consideration for the treatment of PD. They aim to be economical and to offer long-term relief without adverse events. These treatments could be viewed as competing with cell therapies, yet synergies between them could be explored. For example, as cell therapies are purely focused on symptomatic management through the restoration of dopaminergic activity, they could easily be combined with a disease-modifying therapy. Such approaches may offer the opportunity to both restore and maintain dopaminergic tone in brain tissue affected by PD while slowing cell and synaptic losses at other brain sites.

Alternative symptomatic dopaminergic targeted approaches to cell therapy are also being considered^15–18^. Some of these therapies show promise, yet the rationale for the use of cell-based approaches is built on decades of preclinical and clinical data using human foetal midbrain dopaminergic tissue. In this regard, transplantation of the foetal ventral mesencephalon has been shown to provide long-term clinical benefits in some (but not all) patients with PD. Despite these benefits being mainly restricted to motor control, they are associated with a better quality of life. In addition, foetal grafts have been shown to survive for over 20 years with the restoration of dopaminergic innervation in the grafted striatum, as demonstrated post mortem via positron emission tomography (PET)^19,20,29–31,21–28^. However, not all patients have benefited from these transplants. In some instances, this has been the case even when the grafts have apparently restored striatal ^18^F-dopa PET imaging back to normal^32^. Furthermore, pathology in the grafted tissue has now been shown to be a consistent finding in grafts surviving for more than 10 years, although the extent to which this compromises the transplanted dopamine cells is debatable^33–35^.

In summary, clinical data built on solid preclinical data from the 1980s has shown that foetal dopaminergic allografts can survive long-term in the parkinsonian brain, with functional benefits that generally correlate with improvements seen in dopamine restoration on imaging the transplanted brain. This has even led to some patients being able to stop taking oral L-Dopa completely^36^. The evidence shows that dopamine cell therapies for PD work. Yet, can they be deployed robustly, consistently, and in a scalable and cost-effective manner?

## Stem-cell sources

One of the main limitations of cell-based therapies for PD is that these approaches cannot treat many of PD’s non-motor symptoms because they are driven by pathologies that lie outside the dopaminergic nigrostriatal pathway. It is well documented that PD is a complex neuropsychiatric disorder, affecting not only motor function but presenting with a range of non-motor symptoms that are found in up to 98% of patients. They arise in a heterogeneous manner, differing in phenotype and severity between individuals and include gastrointestinal and autonomic dysfunction, mood disorders, sleep disturbances and cognitive impairment among others^37,38^. It is thought that this is due to pathology across a range of sites in the central and enteric division of the autonomic nervous system^39,40^. With the focus of cell therapies on restoring dopaminergic striatal innervation, many have theorised that the non-motor symptoms of PD will persist after this treatment and continue to worsen as the disease progresses. A study that supports this theory followed 3 patients 13-16 years after receiving foetal ventral mesencephalic grafts and showed that although grafting alleviated motor dysfunction, non-motor symptoms such as depression, sleep disorders and visual hallucinations persisted. These were linked to lasting dysfunction in the serotonergic systems that project out of the raphe nuclei^41^. Although non-motor symptoms can have a detrimental impact on patient quality of life (QOL)^37^, the motor symptoms of PD are still thought to be one of the most significant contributors to a loss of patient QOL. This is corroborated by a significant improvement in reported patient QOL after cell transplantation therapy when compared to PD controls^41^.

Another important consideration influencing the effectiveness of cell-therapies on PD is the pathogenic mechanism of the disease. Beyond cell intrinsic alpha-synucleinopathy, the microbiome and neuroinflammation are emerging as key contributors to PD pathogenesis. The link between PD and gastrointestinal dysfunction has been established for over two decades^42^, with a significant number of patients reporting problems such as constipation^43–45^, which often precede the onset of motor symptoms^46,47^. The role of inflammation in PD pathogenesis is also supported by the presence of an increased number of reactive microglia in PD brains^48–50^ as well as disturbances in circulating immune-related markers of the disease^51^. Indeed, it is now thought that these two systems may link directly to one another, as has been shown in a preclinical rodent model^52^, which has led some investigators to look for therapies that simultaneously target both the GI tract and inflammation^53,54^. Because cell replacement does not offer a cure to PD, only palliation of the dopamine sensitive features of the condition, these therapies may also be of merit if combined with cell replacement given that they should act synergistically. Nevertheless, when combined with disease modifying agents targeting the GI tract and inflammation may transform the life of a patient with PD.

To date, PD proof-of-principle studies using allogenic foetal ventral mesencephalic tissue have shown sustained clinical benefit in a subset of PD patients over many years, with evidence of graft survival at post mortem and restoration of dopaminergic innervation in the grafted striatum^27,55–57^ (as discussed above). However, there are major ethical and religious objections to the use of aborted human foetal tissue and even if better, more consistent results could be reproduced by grafting it into patients, the problems of tissue supply would still need to be overcome. Furthermore, restrictions on government funded research using foetal tissue in America means that research and therapeutics pertaining to the use of foetal tissue in PD, however promising, may not be possible in other countries^58^.

Additionally, the need to source multiple foetuses per grafted hemisphere over short periods of time (in order to ensure enough dopaminergic neurons survive transplantation), creates major logistical problems and further limits its widespread adoption along with the inability to standardise the grafted tissue implant^59^. For these reasons, an ethically acceptable, renewable source of stem cells is needed and several have been identified.

So, which stem cell source could be used that fulfil these requirements? The use of embryonic stem cell (ESC) lines is ethically controversial because it involves the destruction of human embryos. Furthermore, precaution is warranted when considering using ESC lines derived from countries that have had cases of the new variant Creutzfeldt-Jakob disease (nvCJD), which comes from the ingestion of Bovine Spongiform Encephalopathy (BSE) infected meat. Although it is worth noting that CJD is a rare disorder, it is a fatal prion disease with no known cure which primarily affects the brain. Moreover, iatrogenic transmission has occurred in cases of blood transfusions, dural grafts, growth hormone therapy and organ donation, so in theory embryonic stem cell (ESC) derived products could transmit the prion if the donor parents were asymptomatic carriers of nvCJD following BSE infection. As there is no way of screening for CJD in vitro, consideration of the source from which a stem cell line is derived is necessary^60^ and underlies the reason why the Food and Drug Administration (FDA) are reluctant to consider human ESC products from cell lines made from donors in BSE affected countries (subsection ‘Embryonic stem cells’).

A further consideration when selecting the optimal starting material is that not all cell lines differentiate consistently into the product of choice and thus searching for the line which differentiates on demand and that will be accessible globally are important considerations. For PD, this has led to research groups using the H9 ES cell line^61^; allogeneic induced pluripotent stem cell (iPSC) lines^62^, the ESC RC17 line^63^ and even autologous iPSC lines^64^, as well as parthenogenetic stem cells^65^ (Table 2).

### Parthenogenetic neural stem cells

Human parthenogenetic-derived NSCs (hpNSCs) are harvested from unfertilised oocytes, thus circumventing ethical concerns associated with embryo destruction. This approach diverges from that of conventional cell replacement therapies, which typically involve grafting committed dopaminergic progenitors. Instead, hpNSCs that are ‘uncommitted’ at the time of engraftment, are used with the expectation that they will produce relevant growth factors or stimulate the host microenvironment or both, which will guide the cells towards a terminal A9 dopaminergic lineage. Therefore, the advantages of this approach are that NSCs are less time-consuming and expensive to manufacture than other cell-based methods while having multiple mechanisms of action. However, while some studies have demonstrated that NSCs are capable of differentiating into dopaminergic neurons in vivo, their conversion efficiency is low and not at a level that is likely to translate into a major clinical benefit for the patient^65–71^.

**Fig. 1:**
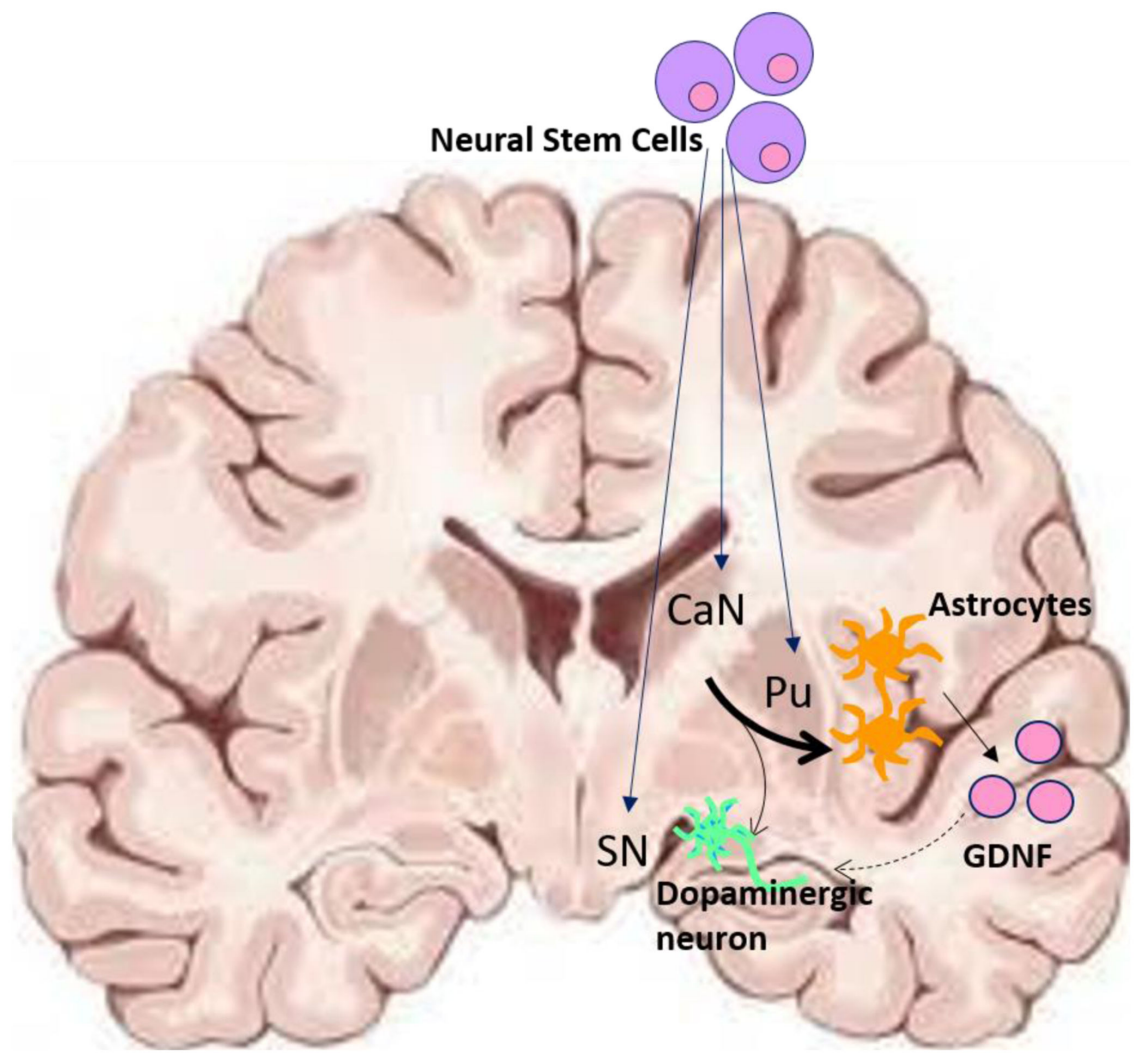
Putative mechanism of action of parthenogenetic neural stem cells for the treatment of PD. Neural stem cells (NSCs) are implanted at multiple sites — the caudate nucleus (CaN), the putamen (Pu) and the substantia nigra (SN) — and allowed to terminally differentiate in vivo. NSCs typically differentiate into small populations of dopaminergic neurons and astrocytes, which may supply neurotrophic support in the form of GDNF to the intrinsic and grafted dopaminergic neurons/fibres. The weight of the arrows provides an indication of conversion efficiencies in vivo. The dotted arrow represents neurotrophic support.

In further support of the clinical adoption of hpNSCs, is the claim that they can provide neurotrophic support for both resident and grafted dopaminergic neurons^66,72,73^. For example, it has been shown that hpNSC transplantation results in some biochemical improvements in the parkinsonian brain, specifically, an increase in neurotrophins--glial cell line-derived neurotrophic factor (GDNF) and brain-derived neurotrophic factor, in and around the injection site in rodents and non-human primates^67,71,73^ (Fig. 1). In some instances, this has also been shown to ameliorate motor deficits in rodent models of PD^72^. Yet nigrostriatal rescue with neurotrophic factors has so far not been shown to work reliably in patients^74–76^. therefore, again, whether NSCs have the potency to rescue neurotrophic support in a clinically meaningful way is debatable. Such concerns are supported by the lack of significant behavioural and motor rescue in animal models of PD using hpNSCs ^65,67,68^. Thus, the main bottleneck pertaining to the use of this cell source, is the lack of preclinical data to suggest that their transplantation offers a realistic chance of clinical benefit to the patient.

**Fig. 2:**
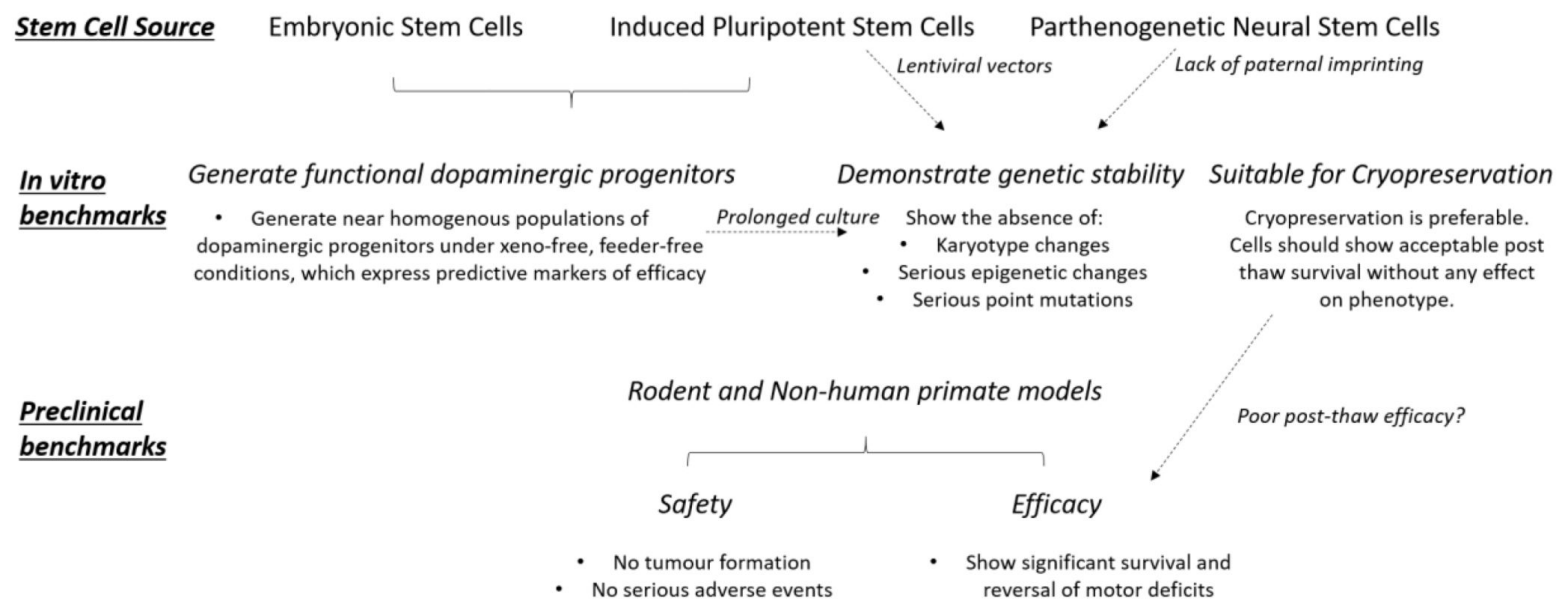
Preclinical benchmarks of efficacy and safety required for a stem cell therapy for the treatment of PD to be considered for a clinical trial. A first requirement is to be able to generate the cell type of choice (for the treatment of PD, A9 dopaminergic progenitors; top). When considering embryonic or induced pluripotent stem cells, the investigators must ensure that these can reliably generate sufficient numbers of functional dopaminergic progenitors expressing predictive markers of efficacy (middle left). All stem cell sources must demonstrate genetic stability. These can be defined as the absence of major changes in karyotype and the absence of epigenetic and/or point variants that could be implicated in relevant disease or in tumour formation (centre middle). It has been argued that, for induced pluripotent stem cells and parthenogenetic neural stem cells, the incidence of genetic aberrations may be increased owing to the use of lentiviral vectors and to the lack of paternal imprinting, respectively. If a stem cell therapy is to be widely used clinically, the ability to cryopreserve the product is preferable. However, cryopreservation must not have any detrimental impact on the viability, phenotype and/or functional efficacy of the cells, post-thaw. Because of the lack of relevant data, the risk of poor post-thaw survival and the effect of cryopreservation on cell phenotype require further investigation (middle right). At the preclinical-assessment stage, safety in cell grafting should be shown. Of upmost importance is that the grafts do not generate tumours; also, they should not result in any other serious adverse events, such as migration and integration into normal neural circuits. Moreover, the regulatory authorities require biodistribution and toxicity data for the cell product. Furthermore, for a product to be considered a viable clinical option, the grafted cells should be able to reverse motor dysfunction in a chemically lesioned rodent (and possibly in a non-human primate model) of PD, and this clinical benefit should be sustained (bottom). The dashed arrows indicate trade-offs between requirements.

Furthermore, due to the nature of oocyte derivation, parthenogenetic stem cells lack paternal imprinting and are thought to retain plasticity. While the significance of these aberrant methylation patterns and the enhanced differentiation potential has yet to be defined, it remains a safety concern, even though the limited published preclinical data suggests that there are no associated major adverse effects^77,78,79^ (Fig. 2).

Despite these concerns, in 2016 in Australia a phase I clinical trial began that has transplanted hpNSCs into 12 patients (ClinicalTrials.gov NCT02452723) (Tables 1 and 2). As with all phase I studies, the primary clinical end-point is to assess the safety and tolerability—in this case--of hpNSC treatment in PD patients. To date, these cells do seem to be safe when grafted into patients^77,80^.

### Embryonic stem cells

One of the main advantages of using embryonic stem cell-derived dopaminergic neurons is that protocols exist which allow for the reproducible and reliable production of large numbers of clinical-grade, near homogenous populations of the correct cell type^81^. A decade of work exploring the development of dopaminergic neurons in vivo and then elucidating how this could be recapitulated in vitro have gone into refining such protocols. One critical study identified that dopaminergic neurons arise from the floor-plate of the mesencephalon, as opposed to the neuroepithelium, where most neuronal cell types originate^82^. This observation subsequently allowed protocols to be developed that recapitulate this in vivo signalling pathway. A second milestone was the concept of ‘Dual SMAD inhibition’, which allowed for the generation of near homogenous populations of neural stem cells^83^ from stem cell sources. This has now led to protocols that recapitulate WNT1-mediated caudalization through the use of a GSK3 inhibitor CT99021^84^,the timed addition of FGF8b^85^ and a ventralizing signal directing cells towards a floor-plate lineage using sonic hedgehog ^86^. Although generating ESC-derived dopaminergic progenitors may be more time-consuming than obtaining NSC’s, directed differentiation from ESCs to dopaminergic progenitors and mature neurons can be achieved in as little as 16 and ~45 days, respectively^81^. As a result, ESC-derived dopaminergic progenitors or neurons or both are an ideal candidate for cell therapies in PD as well as for disease modelling and in vitro drug screening.

Moreover, contrary to preclinical data pertaining to the use of NSCs, multiple groups have demonstrated the potential therapeutic benefit of ESCs for PD. The restorative power of ESC-derived dopaminergic neurons was first demonstrated in 2006 in 6-OHDA lesioned rats^87^. Although this study offered promise, it highlighted a key safety concern associated with any stem cell-based therapy, namely the presence of undifferentiated neural stem cells within the grafts that had proliferative potential. However, with the development of better differentiation protocols, which produce highly homogenous, differentiated phenotypes, these concerns have been minimised. Consequently, many more groups have been able to validate the ability of ESC-derived dopaminergic progenitors to integrate, survive and re-innervate the striatum and by so doing reverse motor deficits in both rodent and non-human primate models of PD, in the absence of tumour formation^84,88,89^. Importantly, one highly significant study has been able to relate the efficacy and potency of ESC-derived dopamine grafts to that of foetal grafts, in preclinical models^90^. This study therefore offers hope that ESC-derived dopaminergic progenitors could work as well as the successful foetal ventral mesencephalic transplants in patients of the correct PD phenotype.

One important consideration for the use of ESCs is, as discussed above, is the choice of the cell line. Some cell lines have an increased propensity to differentiate down certain lineages than others^91,92^. However, some ESC lines have been identified which can be reliably and reproducibly differentiated down an A9 dopaminergic lineage, including the RC17 and H9, both of which are set to be assessed in planned clinical trials (Table 2). Another concern is the origin of the ESC lines. This is important because the FDA currently advise against the use of tissues from BSE affected countries, such as the UK, because we currently have no recommended and robust means for testing for relevant prion proteins in these cell products (see above)^93^. This legislation currently prevents the use of tissues, such as blood, from any individual who may have been in an affected country at the time of the BSE outbreak^94^. Thus, some cell lines cannot currently be used for therapeutic application in the US, unless the FDA can be convinced that there are special circumstances which may be the case for a cell product that is only being used for one condition in more elderly people - such as dopamine cell therapies in PD. Restrictions specific to other countries also cause problems when trying to identify an ESC line that could be accessible to everyone. For example, German legislation prohibits the use of ESC lines that were generated after May 2007, limiting the number of ESC lines that can be used therapeutically^95^. And in Italy, the production of ESC lines is banned, while the use of imported lines is permitted^96^. Therefore, thorough consideration of current legislation must be assessed when selecting an ESC line, especially if the investigators wish to employ this strategy internationally with an eye to the US market.

In addition, as allografts, the introduction of ESC-derived products is likely to elicit some kind of local host immune response and with this comes a risk of graft rejection. This issue is not as straightforward as that encountered with peripheral organ transplants. First, the immunogenicity of ESC derived neuroblasts is not known but is likely to be similar to that seen with foetal tissue which expresses very low levels of major histocompatibility complex (MHC)^97^. Second, the brain is considered a relatively immune privileged site with no professional antigen presenting cells, a blood brain barrier and limited lymphatic drainage. Thus, some studies have suggested that allograft rejection does happen in the CNS^98,99^, while others report that immunogenicity is not an issue with intracerebral allografting^100^. To maximise the chances of graft success in the clinic, most groups working in this area feel immunosuppression is recommended, although this does not need to be life-long, but probably only for 1-2 years post grafting.

Despite the persistence of minor barriers in optimising the translation of human ESCs (hESCs) to the clinic for PD, they are still being tested in trials for this condition (Table 1) (Table 2). In addition, hESC-based approaches for other neural disorders have also been explored and have shown promise(Table 3)^101,102^.

### Induced pluripotent stem cells

iPSCs were first derived from somatic cells in 2006 through the introduction of a quartet of transcription factors, now termed the ‘Yamanaka factors’^103^. This discovery has revolutionised the field of stem cell research and regenerative medicine. However, the primary concern in clinically translating products derived from iPSCs is the use of lentiviral vectors in their reprogramming, as these vectors insert sporadically into the genome and with this comes the risk of tumorigenicity. This is one of the reasons some consider ESCs to be preferable to iPSCs; in fact, one study estimated that iPSCs harbour around 10x more mutations than their fibroblast precursors due to what they deemed ‘reprogramming-associated mutations’. However, it is of note that no transduction-induced tumorigenic potential has been recorded in iPSC-derived dopaminergic progenitors or neurons to date, yet, their post-transduction genotype and karyotype screening will be critical to ensure safety^62,104–108^.

By virtue of their origin, iPSCs can be either allogenic or autologous. One of the main advantages of using autologous iPSCs is that they should be recognised as “self” by the host and by so doing obviate the need for co-administrative immunosuppression^108^. Some consider this one of the main advantages of using autologous iPSCs in favour of allogenic or ESCs. However, the lack of immunogenicity of autologous tissues remains contentious as studies have shown that autologous iPSC-derived products may still elicit immune responses in the host^109^. The other concern with using autologous cells is that you are grafting back cells derived from a host that developed the disease in the first place. In PD it is known that unrelated human foetal ventral mesencephalic tissue can acquire the pathology of PD after a number of years^33,34^. Furthermore, one study has demonstrated the potential for ESC-derived dopamine grafts to acquire alpha-synuclein pathology^110^. Thus, in theory, acquired pathology may happen more readily with autologous iPSC-derived dopamine cells and by doing so, compromise the transplant. In addition, there are currently economic and practical constraints accompanying the autologous approach. Inter-individual genetic variation is concordant with batch heterogeneity, necessitating reprogramming, adaptation of the differentiation culture protocol and the use of non-standardised progenitor cultures, many of which may need to be abandoned due to unsuitability^111–113^. For example, in a RIKEN trial using autologous iPSC-derived retinal pigmented epithelial cells for the treatment of age-related macular degeneration. Furthermore, the cost of such procedures is estimated at around £1-2million per patient given the extensive safety testing that would currently have to be done for every cell line (that is, per patient)^114^. In spite of such barriers, it has been shown that PD patient tissues can be used to generate autologous iPSC-derived dopaminergic progenitors and neurons^62,104,107,108^. Importantly, it has also been demonstrated that there is no significant difference between cells derived from healthy individuals and PD patient-derived iPSCs and their subsequent dopaminergic progenitors and neurons when grafted xenogenically into non-human primates^62^. Indeed, patient-derived iPSCs have been shown to survive, re-innervate the striatum and relieve motor dysfunction in the absence of tumour formation, in both rodent and non-human primate models of PD^62,104,107,108^. One longitudinal study demonstrated sustained clinical benefit in non-human primates for up to 2 years^104^. Furthermore, earlier this year, investigators reported the safety of autologous iPSC-derived dopaminergic progenitors, engrafted into the putamen of a 69-year-old idiopathic PD patient. The results of this study suggest that cells can survive without immunosuppression for up to 2 years. The survival of patient-derived iPSC-derived dopamine cells was also found to be concomitant with some symptomatic improvements. However, the most striking improvement pertained to an overall increase in patient QOL^115^. Although preliminary and only in one patient with no change in functional dopamine imaging using PET, these results show promise for the future of autologous iPSC-derived dopaminergic cell therapies. Given this promise, it is not surprising that another clinical trial using autologous iPSC-derived dopaminergic progenitors for PD is currently being planned^64^. The results of both studies combined may make for a strong argument in favour of this specific approach.

However, due to the economic and practical constraints, which can be associated with autologous iPSCs, there is an increasing emphasis on the use of allogenic iPSC products. Because one of the main limitations of allografts is their immunogenic potential, which is less of a concern for autologous grafts^116,117^, there is an increasing emphasis on the identification of strategies to reduce their immunogenic potential or immunogenically match to the patient or both. One such method involves generation of an iPSC bank consisting of many allogenic cell lines that cover the host population MHC repertoire. Host-donor MHC matching has been shown to significantly reduce graft immunogenicity and increase graft survival, with Tacrolimus monotherapy sufficient to dampen immune rejection and enhance graft survival in animal models in both MHC matched and mismatched cases^116^. Although HLA matching can be an effective mechanism of reducing immunogenicity, HLA matched donors are not always identifiable, especially when considering mixed race hosts^118^. To circumvent this issue, other groups are focusing on the generation of a universal iPSC line, genetically modified to not express MHC complexes. Indeed, proof-of concept studies have demonstrated that this is possible whilst not adversely affecting the differentiation capacity of allogenic human and mouse iPSCs^119–121^ (section ‘Off-the-shelf therapies’). Although preliminary, the option to modify MHC class may render the necessity for MHC matching redundant and minimise allograft immunogenicity. However, it should be realised that any gene editing of a stem cell may have off-target effects which will need to be carefully looked for when one is genotyping the cell line and its product (section ‘Off-the-shelf therapies’). Thus, at present, any study hoping to use allogenic iPSCs will have to rely on conventional immunosuppressant regimens to reduce the chance of graft rejection.

In spite of these barriers, reliable protocols now exist which allow for the generation of large numbers of near homogenous populations of iPSC-derived dopaminergic progenitors and neurons^81^. Therefore, in much the same way as ESCs, the availability of these cells makes them one of the most suitable candidates for cell replacement therapies. Furthermore, there is a robust repertoire of preclinical data to support the efficacy and the potency of allogenic iPSC-derived dopaminergic progenitors. First, allogenic iPSC lines have been shown to reliably differentiate into dopaminergic progenitors and functional, mature neurons^106,122,123^, with a conversion efficiency mirroring that of hESCs^81,124^. Second, these cells have been shown to alleviate motor dysfunction in animal models of PD after engraftment, with no tumour formation^62,106,116,122,123^, with one study demonstrating the efficacy of allogenic iPSC-derived dopaminergic grafts in non-human primates for 2 years^62^. The post mortem analysis of this study also confirmed that these cells were able to survive, integrate and innervate the host striatum^62^. On the basis of this, a clinical trial to assess the safety of iPSCs derived dopamine transplants in moderate PD patients is now underway in Kyoto, Japan^125^ (Table 1) (Table 2), with the first patient having been grafted in 2018. iPSCs are also being considered as regenerative therapies for a range of other neurodegenerative diseases (Table 3).

## Manufacturing, storage and testing

### Good manufacturing practice

To be suitable for clinical application, all cell sources must be derived and cultured at an appropriate GMP level, with evidence of safety in vivo. The exact requirements for safety studies varies depending on which agency is being approached, for example, the Medicines and Healthcare products Regulatory Agency (MHRA) versus FDA. However, this typically involves relatively large numbers of male and female rodents being grafted and followed for many months and studied for tumour formation as well as the biodistribution and toxicity of the cells at a good laboratory practice level.

At the present time, there are several established protocols for the generation of research grade stem cell- derived dopaminergic neurons^83,88^. However, these research grade cultures often use undefined basement membrane matrices, feeder cell populations and xenogeneic culture mediums, all of which should not be used in the clinical setting. Consequently, clinical grade production necessitates feeder-free and xeno-free culture conditions, but this can negatively influence the differentiation capacity and viability of cultures, which has led to inefficient differentiation in some instances^126^. Subsequently, there has been an increasing effort to identify xeno-free adherence matrices and culture media, supplemented with small molecules which recapitulate preferential signalling interactions provided by feeder cells. Taken together this has led to the identification of effective xeno-free media and matrices, which has aided in the generation of protocols for the efficient production of clinical grade stem cell-derived dopaminergic neurons^81,84,127^.

### Manufacturing and storage

In order for any cell product to be suitable for large-scale clinical application, it will need to be manufactured in a reproducible way that allows for its scale-up and scale-out. When short and efficient differentiation protocols exist, this is not an issue- for example, with dopaminergic neurons from human induced pluripotent stem cells. However if the process is a lengthy and complex one then this can be a big problem- for example, some protocols for making glial cells^128^.

The manufacturing of cells can involve the use of bioreactors or other devices enabling expansion of the cell product to the numbers needed for clinical use. This has also been looked at to some extent with dopaminergic neurons from stem cell sources using automated microfluidic devices. These organ-on-a-chip systems require a fraction of the reagents necessitated by macroscopic cultures and have built-in software to batch test cultures^129,130^. Such bioreactors are designed with the aim of recapitulating the PD microenvironment with the intention of improving in vitro models of disease rather than a therapeutic agent, yet it is not unreasonable to assume they could be used in this capacity. However, microfluidic cell culture methods inherently generate a lower number of Tyrosine hydroxylase positive neurons (~20% vs ~90% using typical in vitro culture) due to lower starting culture densities and low conversion efficacy compared to typical in vitro cultures. Furthermore, such methods have not been tested or optimised for commonly used cell lines or generated cells in the numbers needed for clinical translation. Thus, currently, automated cell differentiation has yet to prove itself as a reliable way forward, but as the field matures so might this approach. In the interim, given the efficiency of the current differentiation protocols (16 days to differentiate the cells ready for transplantation)^81^, manual differentiation is still a viable option.

In addition, the cell product ideally should be appropriate for cryopreservation. Historically, iPSCs and ESCs have proven refractory to cryopreservation, displaying poor post-thaw recovery and differentiation^131,132^. Typically, this is attributed to the formation of intracellular ice crystals and high solute concentrations; therefore, measures, which minimise the occurrence of such events, are thought to be beneficial to post-thaw viability and growth. Such strategies have now been shown to increase the efficiency of post-thaw recovery, reaching up to 80%^81,133–136^. Despite this progress in survival, it is imperative that the cryopreserved progenitors not only survive but retain their functional efficacy. Cryopreservation is thought to impose both physical and molecular stress on cells and has been speculated to be the reason for negative outcomes in a clinical trial using cryopreserved mesenchymal stem cells^137^. Despite this, to date, the preclinical efficacy and safety of cryopreserved stem cell-derived dopaminergic progenitors has been demonstrated in some studies^134,135^. Therefore, more data is necessary pertaining to the effect on in vivo functionality and safety of cryopreserved progenitors. Furthermore, there are practical considerations when reviving a cryopreserved product for clinical use, which relates to whether the thawing and washing of the stored cell product constitutes part of the manufacturing process, as this brings with it requirements for the use of GMP facilities. This could therefore pose additional problems for any product wishing to be trialled or used across many clinical sites as not every hospital has a GMP facility.

### Safety testing

There are multiple factors involved in the process of converting stem cell lines into clinically suitable A9 dopaminergic neuronal progenitors. The transition from a research grade culture protocol to one which is GMP compliant can significantly impact on the final cell product. This was highlighted in one study using a neural precursor cell in Alzheimer’s disease, where the GMP grade product displayed increased tumorigenicity and inefficacy in comparison to an equivalent, earlier, research grade cell product^138^.

A major key element in the assessment of the safety of a cell line and its product is the genetic variance that it possesses and its significance, which will vary as a function of passage number and time in culture^139^ (Fig. 2). This raises challenges as to what constitutes a detrimental genetic variant and how one can detect such variance in a large population of cells. Currently, multiple strategies exist to determine the genomic identity of stem cell-derived progenitors with varying levels of sensitivity (Table 4). A survey conducted in 2011 based on 125 human ESC-lines reported that chromosomal alterations were the most common abnormality seen with prolonged culture. Trisomies of chromosomes 1, 12, 17 and 20 were common and are thought to imbue the line with a selective growth advantage^140^. Karyotype aberrations are often screened for prior to the use of a cell line for therapeutic purposes and can be detected relatively easily through conventional cytogenetic analysis, but only if their prevalence in culture exceeds 6-10%^141^.

However, even though karyotypic aberrations are relatively easy to detect, such changes are not the only type of non-random mutations associated with stem cells and their culture. Certain copy number variants and point mutations are also associated with stem cell culture^141^. One specific example is a copy number variant on chromosome 20, thought to be present in around 18-20% of ESC and iPSC lines^140,142^. This variant is thought to incur a selective growth advantage through the amplification of the anti-apoptotic protein, BCL-XL^143^. In addition, non-random epigenetic changes warrant consideration. One study demonstrated the recurrent hyper-methylation of a known tumour suppressor gene - TSPYL5 which has been shown to play a critical role in multiple cancers^144^.

Therefore, increasingly sensitive strategies or complementary methods to karyotyping are required. qPCR can be used to assess targeted variants at the level of single genes and may subsequently be used as a complementary method to investigate polymorphisms in high-risk genomic regions. Despite this, our understanding of the relationship between genetic variance and prolonged stem cell culture is relatively naive, raising issues surrounding the choice of targeted genes for assessment. Therefore, until these are defined, two strategies present themselves; reduce in vitro acquired genetic variance or use increasingly specific techniques such as whole genome sequencing with well-defined criteria for excluding cell lines with certain genetic variants.

Indeed, whole-genome sequencing can detect deviations at the single gene level such as copy number variants and single nucleotide variants, undetectable by karyotyping, but what exactly this means is often harder to define. However, if non-synonymous aberrations are detected in oncogenes, tumour suppressor genes or genes associated with disease states, neuronal function and development, then there are concerns about proceeding with that line and product or both to clinic^145^.

### Functional testing

The production of dopaminergic neurons from stem cells requires culture conditions that re-create those that govern normal embryonic development towards a ventral mesencephalic lineage. Embryogenesis and gestation are spatiotemporal processes, dependent on the time and concentration at which stem and progenitor cells are exposed to specific growth factors and other small molecules. Many protocols rely on early “dual SMAD inhibition” to promote ectodermal differentiation^146,147^ in combination with additional patterning factors which specify differentiation towards a caudal ventral midbrain lineage^81,84,148^. Therefore, to produce high quality progenitors, it is essential that care is taken to administer adequate amounts of small molecules at appropriate times.

For any cell product, it is important that predictive markers of efficacy can be identified prior to grafting as a means of improving the likelihood of functional benefit. Predicting in vivo efficacy is challenging because, in the case of stem cells for PD, the grafted product needs to differentiate into dopamine cells post grafting which can take many months. Thus, predictive markers are needed at the time the cells are implanted. Such markers have been identified as those that are characteristic of immature midbrain dopaminergic neurons - FOXA2/OTX2/LMX1A^88,148,149^. However, it has been shown that these markers are also present in a non-dopaminergic population of cells, and thus additional, specific markers have been suggested including EN1, CNPY1, SPRY1 and WNT1^63^. As a result, immunostaining and qPCR to check for their presence in progenitor cell products at the time of grafting may be more predictive of a functional dopaminergic transplant.

While these markers can be used to predict the efficacy of dopaminergic cell transplants, it is well documented that only a small proportion of transplanted cells survive and only around 5-54% of these mature into Tyrosine Hydroxylase positive (TH^+^) cells^62,63,84,88,106,150^. One study has reported that ESC-derived dopaminergic progenitors differentiate into both neuronal and non-neuronal cell types, mainly into neurons, astrocytes, and surprisingly vascular leptomeningeal cells^151^. In spite of this, studies have shown that only a relatively small number of TH+ cells are necessary to reverse motor dysfunction in rodents, with one study showing functional recovery in neurotoxic lesioned rats with around only 500 surviving human TH^+^ cells^150^. Although the markers discussed previously have allowed us to better understand how cell composition can affect graft efficacy, we do not yet know the mechanisms by which some cells confer survival advantages over others. When cells are grafted, there are a number of stressors that could lead to cell loss; these include surgery-induced inflammation and host immune rejection. Among these factors, it is important to note that the microenvironment into which these cells are placed is very different to that in which they are cultured. The properties of certain cells and the mechanisms by which these govern survival in the presence of such stressors is as of yet undefined. Studies have previously relied upon the presence of known markers to answer this question. For example, one study reported that CORIN+/Nurr1+ cells showed significantly greater survival than their negative counterparts^106^. However, to truly understand this mechanism, we may need to employ alternative, more sensitive techniques such as single cell RNA sequencing, as used to detect markers of efficacy^63^. If we can employ such techniques and use them to determine how cell composition influences survival, we may be able to answer such questions in the future.

The next question that arises is how one can monitor the grafted cells within the brain and the most obvious way is through imaging - especially given that currently this is the only way possible in patients.

Preclinically, imaging has employed a number of different approaches. While functional Magnetic Resonance Imaging (fMRI), Positron Emission Tomography (PET) and Optical Coherence Tomography (OCT) are valuable clinical prognostic tools, alternative high-throughput, lower cost methods are often used in the preclinical space. These can involve bioluminescence imaging, as a method of determining graft survival and size^152,153^. Notably bioluminescence imaging can also be implemented as a means of identifying proliferation and migration in grafted populations, to establish the safety of such therapies in animal models. However, bioluminescence imaging is currently not used for clinical translation due to the use of lentiviral vectors, responsible for the constitutive expression of luciferase or alternative fluorescent proteins within grafted tissues, and their sporadic insertion into the genome^154^. This not only poses safety risks but may also compromise the integrity and differentiation potential of the grafted cells^155^. Furthermore, despite its efficacy, bioluminescence imaging can be limited by low sensitivity and restricted tissue penetration, especially when using short wavelength emitting substances such as green and red-fluorescent reporters in deep tissues. To circumvent this, many groups are focussing on the production of near-infrared reporter genes, which display increased tissue penetration^156^. Alternative current approaches are focused on the development of multimodal imaging platforms, combining bioluminescence imaging with increasingly sensitive techniques such as MRI. Such bi-modal imaging platforms could present a comprehensive method for assessing the safety and efficacy of cell-based therapies in preclinical models^157–160^.

In clinical trials, graft efficacy is mostly determined through a battery of cognitive and neurological assessments, such as the Unified Parkinson’s Disease Rating Scale examination in PD. However, in the context of cell-based approaches, clinical recovery often emerges sometime after improvements seen on imaging. Therefore, investigators are reliant on neuroimaging techniques as predictive markers of dopaminergic differentiation. PET has primarily been used to study the dopaminergic cells within the transplant and their innervation of the neighbouring striatum. The most commonly used ligand and approach is ^18^Fluoro-Dopa positron emission topography (^18^F-Dopa PET) to look at dopaminergic nerve terminals within the graft^19,22,23,161^. However, there are additional ways to study such grafts including fluorodeoxyglucose (FDG)-PET and fMRI to look at circuit reconstruction^162,163^ as well as structural MRI looking at the graft site for evidence of haemorrhage or cell proliferation- which will be a major issue when stem cell transplants move into patients^150^. These approaches not only confirm cell survival and differentiation into an appropriate phenotype but often precede observable clinical benefit which allows for trials to progress at a rate that is not necessarily dependent on clinically manifest effects.

Ultimately, the functional efficacy of the cell product is a key issue (although perhaps surprisingly not for regulators, at least in early clinical trials). The choice of model used to assess the functional benefits of the cell product is somewhat dependent on what disease is being studied, all of which have limitations when it comes to modelling this in animals. The most commonly used models in PD are chemically lesioned animals. Commonly, rodents and less commonly, non-human primates are exposed to acute doses of neurotoxins, namely 6-hydroxydopamine (6-OHDA) or 1-methyl-4-phenyl-1,2,3,6-tetrahydropyridine (MPTP), which incur motor dysfunction through the loss of the A9 dopaminergic neurons^164^. While these models do not mimic the pathogenesis of PD, they do replicate the dopaminergic loss that one is trying to treat with the cell therapy. Alternative approaches more accurately recapitulate PD pathogenesis by systematic or intracerebral injection of recombinant α-synuclein fibrils^165,166^. Which model one chooses to use depends on the aims of the experiment. For example, when choosing between rodent and non-human primate models, the close anatomical resemblance between non-human primates and humans is an obvious advantage. However, such models are usually reserved for therapies nearing clinical trials due to high cost and more stringent ethical considerations linked to the use of non-human primates^167^. The efficacy of cell therapies in any of these models is generally based upon their ability to restore motor function, which is determined through a battery of functional tests. The alleviation of non-motor symptoms is generally assessed by changes in social and behavioural characteristics^168,169^, however, how any of these relate to patient symptoms and signs is largely unknown.

At a minimum, the assessment of the functionality of dopaminergic neurons should involve grafting cells into the 6-hydroxydopamine (6OHDA) lesion rat model of PD and showing long-term survival, differentiation, integration and complete restoration of behaviour, such as drug-induced rotational behaviour. Potency should be similar to that seen with human foetal dopamine cells (for example, complete reversal of drug-induced rotation with only a few 100 dopaminergic neurons) (Fig. 2).

## Off-the-shelf therapies

Off-the-shelf therapies offer a one-size-fits-all solution. In principle, an off-the-shelf ‘universal’ cell therapy would be derived from one allogeneic master cell line, prepared, quality-controlled and cryopreserved until needed. The main advantage of an off-the-shelf approach is availability: cells would be ready for use as required, which is particularly pertinent in debilitating neurodegenerative diseases and acute onset disorders such as spinal cord trauma and stroke. Also, the use of a defined allogenic stem cell line is increasingly pragmatic, as it diminishes any variability in cell culture caused by inter-individual heterogeneity^170^. Such issues were highlighted by a 2015 RIKEN trial, which aimed to use autologous iPSC-derived retinal-pigmented epithelial cells to treat patients with age-related macular degeneration. However, six mutations, suspected to have been generated during reprogramming and culture, were identified in one of the patient’s cell lines. One of these variants was found to be listed as a cancer somatic mutation, and as such the trial was suspended^113,171^.

Although the central nervous system is considered a relatively immune privileged site, allografts of stem-cell- derived products placed into the central nervous system have shown susceptibility to immune-mediated rejection^99,172^. Hence, immunosuppressive therapies are recommended for use post-transplantation. However, prolonged immunosuppressive regimens have been associated with the increased risk of certain cancers^173,174^ and infections^175^. Therefore, there have been efforts toward the modification of the cell product as a means of reducing immunogenicity. This strategy mainly encompasses adapting the cell product in order to diminish the recognition of allogenic materials by host cytotoxic T cells. Such efforts have largely focused on the knock-out of the major histocompatibility complex (MHC) molecule, which allows host T cells to identify donor tissues as foreign material^176^. A few methods for achieving this have been pursued, mostly involving knock-downs of the whole MHC1 complex or its constitutive parts (such as beta-2-microglobulin^120^). Despite success, a lack of expression of MHC1 can induce cell death through recognition by natural killer (NK) cells, although this effect can be diminished by the inhibitory interaction between HLA-E and CD94/NKG2A surface complexes on NK cells^177^. Some efforts have thus focused on developing allogenic stem cells that constitutively express HLA-E^178^. Other work focused on the modification of the cell product so that it expresses CTLA-4-Ig (cytotoxic T lymphocyte antigen-4-immunoglobulin) or PD-L1 (programmed death-ligand 1), or both. Complementary binding of CTLA-4-Ig and PD-L11 to CTLA-4 and PD-1 on T cells modulates the host immune response, diminishing T-cell activation and proliferation^179^ (Table 5).

## Clinical-trial design

There are many therapies that target the dopaminergic aspects of PD, including conventional drugs as well as neurosurgical interventions (such as deep brain stimulation) and enteral dopaminergic treatments (in particular, DuoDopa). Therefore, for a dopamine cell therapy to be clinically successful, it would have to show equivalence of effects with these strategies at a cost-effective price. Associated with this is identification of the optimal patient group, which has been an issue in some of the already completed trials of foetal ventral mesencephalic tissue^22,161^.

Translating a cell therapy to a clinical setting involves the design of trials that have a reasonable chance to prove efficacy and safety as well as competitive value. Early engagement with licensing agencies and clinicians is thus paramount to define the population of patients that would optimally represent the treatment group. The optimal treatment group would appear to be younger patients with PD, with mild or moderate disease, and without major non-motor problems nor significant L-dopa induced dyskinesia^180^.

## Outlook

It would seem that ESCs, autologous iPSCs and allogenic iPSCs are all suitable candidates for cell-based therapies for PD. All three types of cell have shown promise in a number of preclinical animal models, and the similarity in potency and efficacy observed for these stem cell sources and for foetal tissues offers hope that the efficacy and safety will translate into humans. However, there are concerns about how cellular composition influences the survival of the grafts. In much the same way as characteristic markers of efficacy have been identified, the cellular basis of why a small subset of grafted cells survives in favour of other cellular subsets needs to be investigated. If the genetic underpinnings of this survival advantage can be established, the way in which cultures are screened before grafting could be improved, and grafting a much smaller and more homogeneous and possibly safer population of cells might become possible.

With respect to the use of ESCs, the use of iPSC-based therapies can be associated with an increased risk of significant mutagenesis. However, the genetic integrity of every cell line intended for clinical use must be rigorously assessed. Variance at the genetic, karyotype and epigenetic levels are commonly observed in both ESC and iPSC lines, yet their significance is often unknown. Therefore, whether the techniques employed to establish genetic integrity are sufficiently sensitive and comprehensive to justify clinical use, as exemplified by the RIKEN trial, should be assessed.

If cell therapies are to become mainstream, scalability considerations become paramount. There are reproducible and robust protocols for making dopamine neuroblasts from stem cell sources, which are essential for any clinical development of the product. Yet how these cells can be optimally stored and cryopreserved needs further investigation. The limited amount of data available seems to suggest that cryopreservation is not a major bottleneck in the use of cell-based therapies. Still, there are logistical limitations (whether the thawing and preparation of the cells is deemed to be part of the manufacturing process, and if so whether this will need to be done in GMP facilities) in relation to where the cells can be stored.

Trial design will also be central. As exemplified by clinical trials using foetal ventral mesencephalic tissue, it is difficult to determine the reason for disparities in their outcomes when there are substantial different in their design and execution. The silver lining has been that there is more clarity regarding which instruments can be used, how many cells need to be grafted and at which stage of maturity, and which patient cohorts are most likely to benefit from the therapies. Still, there are remaining challenges associated with cell dosing as well as deposit number and site, as well as with the extent and duration of any immunosuppression. The hope is that greater harmonization across studies will make them more comparable. In this regard, a global initiative (GFORCE-PD)^59,181^ for coordinating stem cell dopamine therapies for PD has been established.

Foetal tissue can substantially improve the lives of some patients with PD. The future of therapies for the treatment of PD will largely be determined by whether the efficacy of foetal-tissue therapy can be consistently recapitulated in clinical trials of stem cell therapies.

## Figures and Tables

**Table 1:** Milestones in the development of stem-cell-based therapies for PD.

Year	Cell type	Event
1988	Foetal ventral mesencephalon	First human transplant.
1998	ESCs	Derivation of human ESCs.
2007	iPSCs	First generation of iPSCs.
2008	ESCs	Generation of the first protocol outlining good differentiation into dopaminergic neurons from human ESCs.
2010	iPSCs	First generation of A9 dopaminergic neurons from iPSCs.
	iPSCs	Patient-derived iPSC-derived dopaminergic neurons reduce motor dysfunction in a 6OHDA rat model.
2011	ESCs	Human ESC-derived dopaminergic neurons reverse motor dysfunction in a rodent 6OHDA model.
2014	ESCs	Proof of principle that human ESC-derived dopaminergic progenitors are safe and efficacious in animal models of PD.
2015	Foetal ventral mesencephalon	First TRANSEURO patient grafted.
	Parthenogenetic NSCs	Proof of concept of NSCs in animal models.
2016	Parthenogenetic NSCs	First patient grafted in the ISCO trial.
2017	Parthenogenetic ESCs	First patient grafted with parthenogenetic ESC-derived neural precursors in the Chinese Academy of Sciences trial.
	iPSCs	Proof of principle that iPSC-derived dopaminergic progenitors are safe and efficacious in animal models of PD.
2018	Foetal ventral mesencephalon	Last TRANSEURO patient grafted.
	iPSCs	First CiRA patient grafted with allogenic iPSC-derived dopaminergic progenitors.
	Parthenogenetic ESCs	Publication of proof-of-principle data pertaining to parthenogenetic neural precursors in PD.
2019	Parthenogenetic NSCs	Last patient grafted in the ISCO trial.
2020	iPSCs	First report of safety in a patient grafted with autologous iPSC-derived dopaminergic cells.
In the near future	iPSCs	Start of the SUMMIT for PD Phase-I clinical trial to assess the use of human autologous iPSC-derived dopaminergic progenitors.
	ESCs	Start of the European STEM-PD and NYSTEM-PD Phase-I clinical trial to assess the use of human embryonic stem cell-derived dopaminergic progenitors.

**Table 2:** Ongoing and planned clinical trials of cell therapies for the treatment of PD. TBD, to be determined.

Trial	Transeuro^182^ NCT01898390	ISCO^183^ NCT02452723	CiRA^125^	Chinese Academy of Sciences^184^ NCT03119636	American multicentre case report^185^	Summit for PD (in set up)^59^	NYSTEM-PD^186^ (in set up)	European STEM-PD^187^(in set up)
**Cell source**	Foetal ventral mesencephalon^a^	Parthenogenetic neural stem cells^b^	Allogenic iPSCs^c^	Parthenogenetic embryonic stem cell derived neural precursors^d^	Autologous iPSCs^e^	Autologous iPSCs^f^	H9 embryonic stem cells^g^	RC17 embryonic stem cells^h^
**Cryopreservation of the cell product**	No	Yes	No	Data not available	Yes	Yes	Yes	Yes
**Screening of cell product for genetic variance/quality control**	None	Yes, flow cytometry and RT- PCR to assess markers of pluripotency.	Yes, sequencing for genes of interest	Data not available	Whole genome sequencing	Whole genome sequencing	Yes, Karyotype analysis by G- banding. Viral testing. Genetic testing TBD.	Yes, flow cytometry and RT- PCR to assess markers of pluripotency and midbrain dopaminergic progenitors. Genetic testing TBD
**Functional testing**	Structural and functional MRI ^18^F-Dopa PET ^11^C-PE2I PET ^11^C-DASB PET	MRI ^18^F-DOPA PET	MRI ^18^F-FLT-PET	MRI DAT-SPECT	^18^F-DOPA PET MRI CT scan	^18^F-DOPA PET DAT- SPECT ^18^F-FLT PET	^18^F-DOPA PET ^11^C-PE2I PET MRI	MRI ^18^F-DOPA PET ^11^C-PE2I PET
**Immunosuppression**	Cyclosporine, Azathioprine and Prednisolone	Yes, but unclear	Tacrolimus	Data not available	None	None	Tacrolimus, mycophenalate, basiliximab, prednisolone.	TBD
**MHC Matching?**	No	No	Yes	Two groups - one HLA matched one mismatched	Autologous transplant	N/A	No	No
**Patient cohort**	30-68y/o early- stage PD patients	30-70y/o moderate to severe PD patients	50-70y/o moderate PD patients	50-80 y/o moderate PD patients	One 69 y/o PD patient	45-70 y/o moderate PD patients.	40-70y/o moderate PD patients.	Moderate stage PD patients 40- 70y/o
**Date of first-in-human transplant**	2015	2016	2018	2017	2018	TBD	TBD	TBD

^a^Grafting of foetal ventral mesencephalic tissue has previously demonstrated efficacy in PD animal models and patients^27,55,57,188^. ^b^NSCs grafted into the brains of rodents and non-human primates was safe but with minimal clinical efficacy^65,67,68^. ^c^Clinical grade iPSC-derived dopaminergic progenitors have demonstrated safety and efficacy in rodent and non-human primate PD models^62^. ^d^Clinical grade ESC-derived neural precursors significantly improved motor dysfunction in some MPTP lesioned non-human primates, in the absence of tumour formation for up to 2 years^184^. ^e^PD patient-derived iPSC-derived dopaminergic precursors significantly reversed motor deficits in 6OHDA lesioned rats, without tumour formation^185^. Results were corroborated in one PD patient, who demonstrated clinical benefit up to 2 years after grafting, without the onset of serious adverse events^185^. ^f^Data pertaining to the safety and efficacy of autologous iPSC-derived dopaminergic neurons is underway. 10 patient-derived iPSC lines have been successfully generated^64^. ^g^Clinical grade H9 ESC-derived dopaminergic progenitors have demonstrated safety and efficacy in preclinical PD models^135,150^. ^h^Clinical grade ESC-derived dopaminergic progenitors have demonstrated safety and efficacy in preclinical PD models^84,88^.

**Table 3:** iPSC-based and ESC-based therapies for neurological disorders.

Disease	Cell source	Stage	Key findings
Traumatic brain injury (TBI)	iPSC	Preclinical	A combination of iPSC-derived neural precursor cells and environmental enrichment substantially rescued cognitive deficits in a rat model of TBI^189^.
			Transplantation of iPSC-derived neural precursors improved social behavioural deficit in a mouse model of TBI^190^.
Spinal cord injury (SCI)	iPSCs	Phase I/II	On the basis of the efficacy and safety observed in rodent^191,192^ and non-human primate^193^ models of SCI, a clinical trial to assess iPSC-derived neural precursors is in the pipeline^194^.
	ESC	Phase I/II	Human ESC-derived oligodendrocytes improved motor function in rodent SCI models in the absence of serious adverse effects^195–197^.
			Preliminary clinical outcomes have displayed signs of safety and efficacy (NCT02302157), but follow-up and full clinical data are not yet available^198^.
Macular degeneration	iPSC	Phase I/II clinical trials	On the basis of the efficacy and safety shown after transplanting iPSC-derived retinal pigmented epithelium into rodents and mammals^199^, two Phase-I/II clinical trials are underway to assess the safety of iPSC-derived retinal pigmented epithelial transplants in patients with age-related macular degeneration^200,201^.
	ESC	Phase I/II	The grafting of ESC-derived-retinal pigmented epithelial cells has led to the rescue of some visual aspects in clinical trials^102,201,202^. However, this has not been recapitulated in other clinical studies^203^.
Amyotrophic lateral sclerosis (ALS)	iPSC	Preclinical	iPSC-derived neural precursors have been shown to improve their phenotype in wild-type ALS^204^ and in SOD-1 mutant ALS mice^205,206^.
	ESC	Preclinical	Preclinical studies have shown significant rescue of motor impairment and reduced disease progression in response to the grafting of ESC-derived-astrocytes in a rodent model of ALS207.
			On the basis of preclinical evidence, Phase-I/II clinical trials (NCT03482050 and NCT02943850) are in the pipeline.
Alzheimer’s disease	iPSC	Preclinical	iPSC-derived neural precursors have been reported to improve memory deficits in a mouse Alzheimer’s model^208^.
	ESC	Preclinical	Transplantation of embryonic stem cell-derived neural precursors improved cognition and memory in a mouse Alzheimer’s model^209,210^.
Huntington’s disease (HD)	iPSC	Preclinical	Mouse iPSC-derived neural precursors improved locomotor deficit in a Huntington’s mouse model^211^. This has been recapitulated using patient iPSC-derived neural precursors^212^.
			To reduce the risk of pathology development in the graft, iPSC-derived neural precursors, transgenically modified to express reduced levels of the mutant Huntingtin gene, and wild type iPSC-derived neural precursors, showed a significant reversal of motor deficit in a mouse model of HD^213^.
	ESCs	Preclinical	ESC-derived neural precursors have shown to relieve motor dysfunction and to improve cognitive impairments in a mouse model of HD^214^.

**Table 4:** Common techniques for the genomic assessment of stem-cell-derived tissues.

Technique	Sensitivity	Advantages	Limitations
**Cytogenetics**			
GTG banding^215^	Can detect numerical and structural karyotype aberrations >5Mb	Relatively cost-effective. Capable of detecting most prevalent karyotype aberrations. Can detect both numeric and structural alterations.	Low resolution (larger than 5Mb). Only mitotic cells can be assessed. Not sensitive enough to detect mosaicism if prevalence is less than 6-10%. Labour-intensive
Fluorescent in situ hybridization^216^	Can detect numerical and structural karyotype aberrations >1-2Mb	Applicable for analysis of mitotic and non-mitotic samples. Higher resolution (1-2Mb) than alternative cytogenetic techniques.	Low specificity for rare, small or complex chromosomal rearrangements. Can only detect aberrations in regions complementary of the probe used. Labour intensive and expensive.
**Nucleic-acid-based techniques**			
RT-PCR^217^	High sensitivity and specificity for the targeted detection of genetic abnormalities	Highly sensitive and specific. High-throughput	Only targeted abnormalities can be detected.
**Next-generation sequencing**			
Whole-exome Sequencing^218^	Defines the genome of exonic DNA regions, allowing the detection of single nucleotide variants, insertions, deletions, copy-number variants and rearrangements	Around a half to a fifth the price of whole-genome sequencing. Highly sensitive (at the single-nucleotide level).	Low throughput Doesn’t detect mutations. Only detects abnormalities in exomic DNA regions.
Wholegenome sequencing^218^	Determines the whole genomic sequencing of coding and non-coding regions of DNA at the single nucleotide base level	Allows detection of mutations in the whole genome. Highly sensitive (at the single-nucleotide level).	High cost Low throughput

**Table 5:** Examples of approaches to modify stem cell products to reduce their immunogenicity.

Method	Rationale	Results	Ref.
CTLA-4-Ig and PD- L1 knock ins in human ESCs.	Stimulation of CTLA-4 and PD- 1 will inhibit CD8+ T-cell activity and proliferation, reducing CD8^+^ T-cell-mediated cytotoxicity.	Tumours formed by the introduction of a CTLA-4-Ig/PD-L1 knock-in human ESCs in a humanized mouse model displayed reduced T cell infiltration and formed larger tumours.	179
Beta-2-Microglobulin knockout	Beta-2-microglobulin forms a constitutive part of the MHC1 complex. Knock-out of Beta-2- Microglobulin inhibits the surface expression of MHC1.	Knock-out cells exhibited ~15% less T- cell activation in PBMC co-culture than wild-type cells^219^. Knock-out cells showed reduced T-cell activation in a mouse model of ischaemic hindlimb^220^.	
MHCI knock-down	Immunogenicity in response to allogenic grafting is primarily consequential on MHC1 T cell activation. Silencing of MHC1 associated genes may inhibit its expression and diminish immune response.	Transplantation of knock-down human ESCs elicited significantly less T-cell activation and survived longer than wildtype cells.	221
Beta-2- Microglobulin and CIITA knock-out	A combined knock out of Beta- 2-Microglobulin and CIITA disrupts the surface expression of MHCI and MHCII, respectively. This would decrease the activation of CD8+ and CD4+ T cells, respectively.	Knock-out cultures shoed a reduced propensity (3-fold) to activate T cells, and produced significantly larger spheroids when co-cultured with peripheral-blood mononuclear cells (with respect to wildtype cells).	222
Disruption of HLA-A and HLA-B expressions with selective retention of HLA-C expression.	Cells non-expressive of MHCI are susceptible to NK- mediated cytotoxicity. Selective HLA-C expression can allow CD8+ T cell evasion while also diminishing cytotoxic NK cell responses.	HLA-C cells evaded both NK and CD8^+^ T- cell-mediated cytotoxicity in vitro and in vivo.	223

## Editorial summary

This Review Article discusses requirements, in cell source, functional and safety testing, manufacturing and storage, and clinical-trial design, for the clinical advancement of cell therapies for Parkinson’s disease.
